# A case report of spindle cell lipoma

**DOI:** 10.1016/j.amsu.2022.103960

**Published:** 2022-06-13

**Authors:** Rema AlRashed, Abdullah Albdah, Feras Alsannaa

**Affiliations:** a General Surgery Resident, Prince Sultan Military Medical City, Saudi Arabia; b Trauma and Acute Care Surgery Fellow, King Saud University, Saudi Arabia; c Trauma and Acute Care Surgery Consultant, Prince Sultan Military Medical City, Saudi Arabia

## Abstract

Spindle cell lipoma (SCL) is an uncommon benign tumor. A 67-year-old male with multiple comorbidities, presented with a complain of swelling in the neck, which was excised, and the diagnosis of spindle shape lipoma was made based on pathological examination.

## Highlights

• Spindle cell lipoma (SCL) is a rare tumor that arises from subcutaneous tissue.
• The definitive diagnosis requires focused gross examination of it varies morphological features is essential, in addition to the Cytological examination.

## 1 Introduction

Spindle cell lipoma (SCL) is an uncommon tumor that arises from subcutaneous tissue, most commonly at the junction between back and neck. SCL frequently manifest in middle-aged male patients [1]. The diagnosis of adipose benign and malignant soft tissue tumors is crucial in order to approach the appropriate management [2]. Bellow We present a case of a 67-year-old male with SCL base on SCARE criteria [3].

## 2 Case report

A 67-year-old comorbid male, presented to the clinic complaining of swelling in the left side of the neck, the swelling was round, soft and mobile and growing slowly in the past two years. He denied any history of pain, discharge, fever, and constitutional symptoms. For past medical history he is known to have diabetes mellitus type 2, Hypertension and dyslipidemia on oral antihypertensives, statins, and oral hypoglycemic agents, Patient also has history of Ischemic heart disease, status post percutaneous coronary intervention, and he's on aspirin since then.

**Fig. 1:**
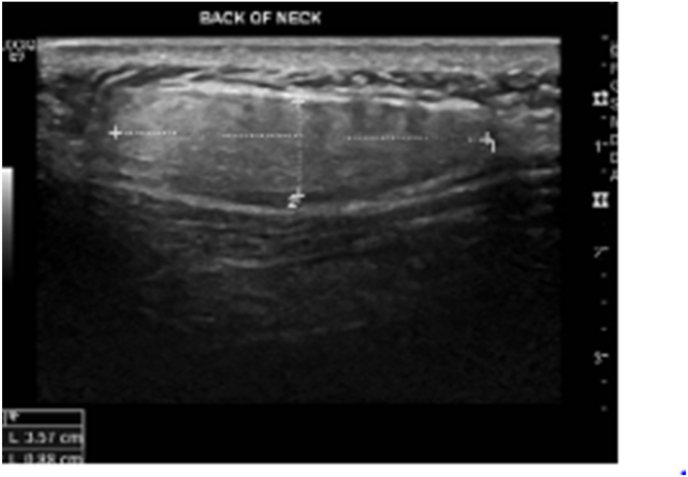
Hypoechoic mass in ultrasound imaging, on the back of the neck.

He denies any family history of related malignancies. On physical examination: there was a palpable soft swelling at posterior aspect of the neck measuring 3 cm in diameter with intact neck and shoulders range of movement. Laboratory investigations including complete blood count, Renal and Hepatic profiles were done and were unremarkable. Furthermore, the lesion was investigated initially with an Ultrasound which demonstrated a 3.6 × 1 cm in size, subcutaneous soft tissue mass (Fig. 1). Differential diagnosis were lipoma and liposarcoma.

**Fig. 2:**
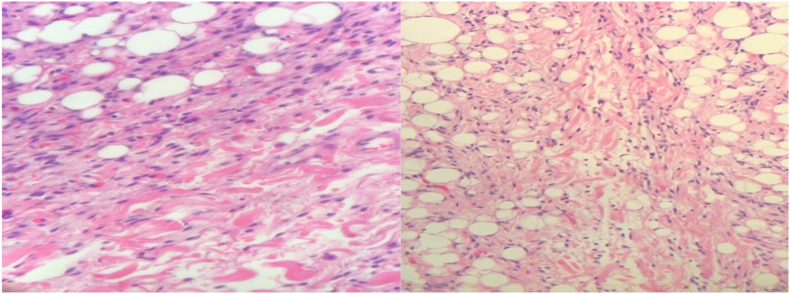
Shows mature adipocytes, collagen bundles and spindle cells.

The option of surgical excision was offered, and an informed consent was obtained The dissection was made around the lesion till it was completely excised including its capsule. The specimen of homogenous yellow cut surface. The specimen was sent for pathological review. Histopathological findings were mature adipocytes collagen fibers and spindle cells, which was consistent of SCL and negative for malignancy (Fig. 2).

The patient was followed up in the clinic 2 weeks post operatively with unremarkable physical examination with the wound being completely healed with no swelling nor recurrence.

## 3 Discussion

Adipose tissue lesions can vary from a benign lipoma to a more serious malignant lesions including liposarcomas [1].

Other subtypes of lipoma may consist of lipomatosis, lipoblastoma, angiolipoma, and myolipoma [4].

SCL is a benign histopathological variant of lipoma [1]. It is manifisted by collagen rich spindle cell taking over adipocytes [5]. SCL are more prevalent in male, especially who are between 40 and 60 yeas-old similar to our patient age group [1]. The most common sites of SCL are posterior aspect of the neck, back, shoulder, other uncommon sites [6].

Moreover, clinical and radiological workoup does not always aid in the diagnosis of SCL. A focused gross examination of it varies morphological features is essential, in addition to the Cytological exam which consists of mature adipocytes and spindle cells with myxoid matrix. In cytogenetic basis it's has been linked to CD34 positivity and often associated with lost 12q ± 16q in karyotyping [7]. On the other hand, The over expression of 12q13-15, may facilitate in diffraction between liposarcoma and SCL [8].

Management of SCL involved a simple excision of the lesion [8]. A Chen, Shuai et al., reported a case that involved 40 cases of SCL where he concluded that excision is considered sufficient, without recurrence in follow up to 8 years [9].

## 4 Conclusion

Spindle cell lipoma is a benign tumor that arises from adipose tissues. It can be diagnosed with microscopic evaluation and managed with excision with good prognosis.

## Provenance and peer review

Not commissioned, externally peer reviewed.

## Ethical approval

Not applicable.

## Sources of funding

No funding.

## Author contribution

Dr. Rema AlRashed: writing the paper, Dr. Abdulllah albudah: review, Dr. Feras sanna: Primary consultant & surgeon final modification.

## Registration of research studies

IT’S A CASE REPORT, NOT A RESEARCH.

Name of the registry:

Unique Identifying number or registration ID:

Hyperlink to your specific registration (must be publicly accessible and will be checked).

## Guarantor

Not applicable.

## Declaration of competing interest

No conflict of interest.
